# Protective Effect of Peptide Calcium Channel Blocker Omega-Hexatoxin-Hv1a on Epithelial Cell during Ischemia–Reperfusion Injury

**DOI:** 10.3390/ph16091314

**Published:** 2023-09-18

**Authors:** Elena Iurova, Eugenia Rastorgueva, Evgenii Beloborodov, Evgeniya Pogodina, Aleksandr Fomin, Dmitrii Sugak, Denis Viktorov, Ivan Tumozov, Yury Saenko

**Affiliations:** 1 Laboratory of Research and Development of Peptide Drugs and Vaccines, S. P. Kapitsa Technological Research Institute, Ulyanovsk State University, 432017 Ulyanovsk, Russia; urovaev523@gmail.com (E.I.); rastorgueva.e.v@yandex.ru (E.R.); beloborodov.evgeniy.a@gmail.com (E.B.); janeg1411@yandex.ru (E.P.); mr.fominan@yandex.ru (A.F.); dmitriysugak@mail.ru (D.S.); viktorov.da@gmail.com (D.V.); ivantumoz@mail.ru (I.T.); 2 Department of General and Clinical Pharmacology and Microbiology, Faculty of Medicine, Ulyanovsk State University, 432017 Ulyanovsk, Russia

**Keywords:** calcium, ischemia–reperfusion injury, peptide toxin

## Abstract

Ischemia–reperfusion injury (IRI) is a common phenomenon that develops both from natural causes and during major operations. Many intracellular processes mediated by calcium ions are involved in the development of IRI. Currently, chemical calcium channel blockers are used but they have a number of limitations. In this article, we study the effect of the omega-hexatoxin-Hv1a peptide toxin, an alternative to chemical calcium channel blockers, on the mechanisms of IRI development in epithelial cell culture. The toxin was produced using solid phase peptide synthesis. IRI was caused by deprivation of glucose, serum and oxygen. The data obtained demonstrate that the omega-hexatoxin-Hv1a toxin in nanomolar concentrations is able to prevent the development of apoptosis and necrosis in epithelial cells by reducing the concentration of calcium, sodium and potassium ions, as well as by delaying rapid normalization of the pH level, affecting the mitochondrial potential and oxidative stress. This toxin can be used as an alternative to chemical calcium channel blockers for preventing tissue and organ IRI due to its low-dose requirement and high bioavailability.

## 1. Introduction

Ischemia–reperfusion injury (IRI) is a common phenomenon that arises both from natural causes (vascular occlusion, myocardial infarction, stroke) and during significant surgical procedures and organ transplantations. All oxygenated tissues are subject to hypoxia and, with normalization of blood circulation, ischemia–reperfusion injury. Despite this, the sensitivity of cells to a hypoxic environment varies among different cell types, but cell deterioration is always proportional to the durability of ischemia. Experimental data indicate that both heart cardiomyocytes [1] and brain neurons [2], as well as liver [3], kidney [4] and lung [5] cells, die during reperfusion.

The progression of IRI is marked by two consecutive events. The initial event is ischemia, which involves the interruption of nutrient and oxygen supply, resulting in malfunction of the electron transport chain within mitochondria. Consequently, this disruption leads to a reduction in adenosine triphosphate (ATP) synthesis, halting the functioning of Na^+^-K^+^-ATPase and Ca^2+^-ATPase on the cell surface. The malfunction of Na^+^-K^+^-ATPase leads to the accumulation of sodium within the cells and the extrusion of potassium outside the cells. Higher sodium levels in cells decrease the activity of the Na^+^-H^+^ pumps (NHE). Ca^2+^-ATPase pumps in the sarco/endoplasmic reticulum also stop functioning, thus limiting calcium reuptake. In cells, the accumulation of hydrogen, sodium and calcium ions causes hyperosmolarity, which leads to the influx of water into the cytoplasm and swelling of the cells. The accumulation of hydrogen leads to a decrease in cellular pH, which in turn impairs enzyme activity and leads to the accumulation of nuclear chromatin. The second stage is reperfusion. It is characterized by the restoration of the flow of nutrients and oxygen. However, the state of the cells, paradoxically, is only becoming worse. Trying to cope with a large amount of H^+^, which were formed at the stage of ischemia, NHE begins to work actively, which in turn further increases the concentration of Ca^2+^ ions by increasing the proton gradient. Meanwhile, Ca^2+^ reuptake by the sarco/endoplasmic reticulum Ca^2+^-ATPase system is also impaired. In an attempt to cope with the huge changes in cytosolic Ca^2+^ levels, transport through the mitochondrial Ca^2+^ uniporter into the mitochondrial matrix is increased. There is a decrease in the concentration of cytosolic Ca^2+^ and an increase in the mitochondrial one, which causes the opening of the mitochondrial permeability transition pore (MPT). The opening of MPT is the end effect in a number of events leading to cell death during reperfusion [6,7].

Cell death can occur during both the ischemic and reperfusion stages, although a higher percentage of cell death is typically observed during reperfusion. The mechanisms underlying cell death in ischemia–reperfusion injuries are diverse and involve various processes such as apoptosis, necrosis, and autophagy [8]. The crucial role of calcium ions (Ca^2+^) in activating multiple signaling pathways associated with IRI has been extensively documented [9,10]. Moreover, recent research has accumulated evidence indicating that the release of Ca^2+^ from endoplasmic reticulum and the influx of Ca^2+^ through voltage-gated ion channels can induce apoptosis [11]. Notably, an elevation in cytosolic Ca^2+^ concentration occurs during both the early and late stages of the apoptotic pathway upon reperfusion. Additionally, high intracellular levels of Ca^2+^ have been linked to cell death through necrosis, while lower levels of Ca^2+^ tend to promote cell death via apoptosis [12].

One common strategy for reducing reperfusion injury is the use of calcium antagonists. These include derivatives of phenylalkylamine (verapamil), benzothiazepine (diltiazem), dihydropyridine (nifedipine), and others. A number of studies have shown that the use of verapamil, diltiazem or nifedipine also alleviate experimental ischemic and reperfusion injury of various organs by reducing cellular permeability to calcium [13,14,15]. However, these drugs have several limitations. These include selectivity with respect to not only L-type channels, but also N- and T-type channels, as well as relatively high doses to achieve the effect [16]. Recently, a research direction associated with the use of peptides as therapeutic molecules for the treatment of IRI has gained significant traction due to their advantageous pharmacokinetic properties, good solubility, as the potential for modifications to enhance their stability and binding affinity [17]. 

In our study, we suggest employing a peptide toxin from arachnid venom as an analogous molecule. This toxin, categorized as a calcium channel blocker, is capable of highly selective binding to target channels, particularly L-type calcium channels in our case. Due to the presence of a special stabilizing structure formed by several disulfide bridges, peptide toxins are able to withstand the effects of enzymes, temperature and the pH level for a long time, which significantly increases their bioavailability. The study of animal toxins as a protective strategy during reperfusion after ischemia has been actively pursued in the literature [18,19,20]. In our study, we propose the use of omega-hexatoxin-Hv1a, a *Hadronyche versuta* spider toxin, and provide data on its effect on cell death and intracellular parameters while simulating ischemia–reperfusion conditions in cell culture. Our hypothesis suggests that the toxin has the potential to mitigate the substantial cell death by facilitating gradual adaptation to the changing metabolism during reperfusion.

## 2. Results

**Figure 1:**
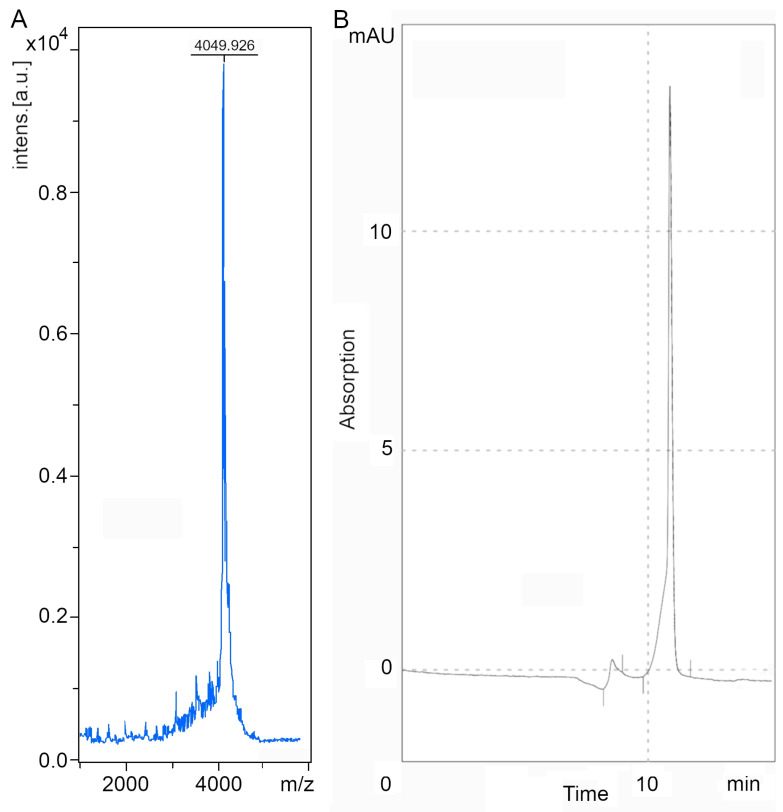
The mass spectrogram (**A**) and chromatogram (**B**) of omega-hexatoxin-Hv1a toxin after synthesis, purification and folding.

As a result of solid-phase peptide synthesis, a toxin with a molecular weight of 4055.015 Da was obtained. Since this toxin belongs to the knottin family and is able to form three disulfide bridges, folding was carried out to achieve the desired structure. After purification and folding, the mass of the toxin was 4049.926 Da and 97.17% pure (Figure 1A,B).

**Figure 2:**
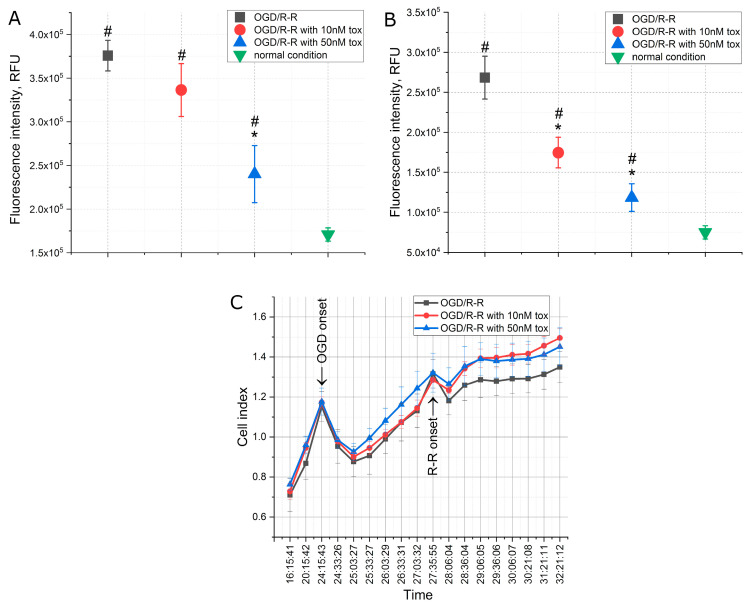
The levels of apoptosis (**A**), necrosis (**B**) and cell index (**C**) when modeling the OGD/R-R conditions in CHO-K1 culture with the addition of omega-hexatoxin-Hv1a toxin (OGD—oxygen–glucose deprivation; R-R—reoxygenation/reperfusion; RFU—relative fluorescence units). (*—*p* < 0.01 when compared with the “OGD/R-R” group, #—*p* < 0.01 when compared with the “normal condition” group).

A number of changes were observed in the intracellular parameters when modeling the conditions of oxygen–glucose deprivation (OGD) caused by a lack of oxygen and nutrients, primarily glucose and serum, for 3 h in a CHO-K1 culture, followed by a sharp restoration of the concentration of oxygen and nutrients (reoxygenation and reperfusion). The addition of the toxin at the reoxygenation–reperfusion stage at different concentrations affected these parameters to varying degrees. As depicted in Figure 2, the period of reperfusion following OGD demonstrated a twofold increase in apoptosis levels and a fourfold increase in necrosis levels (Figure 2A,B). However, upon the addition of omega-hexatoxin-Hv1a toxin, the development of both apoptosis and necrosis was reduced. In the case of apoptosis, the effect manifested itself at a toxin concentration of 50 nM, when the level of apoptosis increased by about 1.5 times higher than normal conditions, and in the case of necrosis, the effect occurred already at a concentration of 10 nM and was dose-dependent. Thus, at a toxin concentration of 10 nM, the level of necrosis increased only by about two times, and at a concentration of 50 nM, it increased by about 1.5 times above normal conditions. 

When analyzing the cell index, which indicates the degree of cell adhesion under the conditions studied, it became obvious that, at the OGD stage, the cells underwent a decrease in adhesion; however, after 1 h, the cells recovered and continued to grow (Figure 2C). At the reoxygenation–reperfusion stage, there was also a decrease in adhesion in the first minutes, but after 30 min, the culture began to recover. However, in the presence of the toxin in both concentrations, recovery occurred in a shorter period of time, in contrast to the control conditions without the toxin. At a toxin concentration of 10 nM, the index returned to the prereperfusion level after 45 min, and the same was true for a concentration of 50 nM.

**Figure 3:**
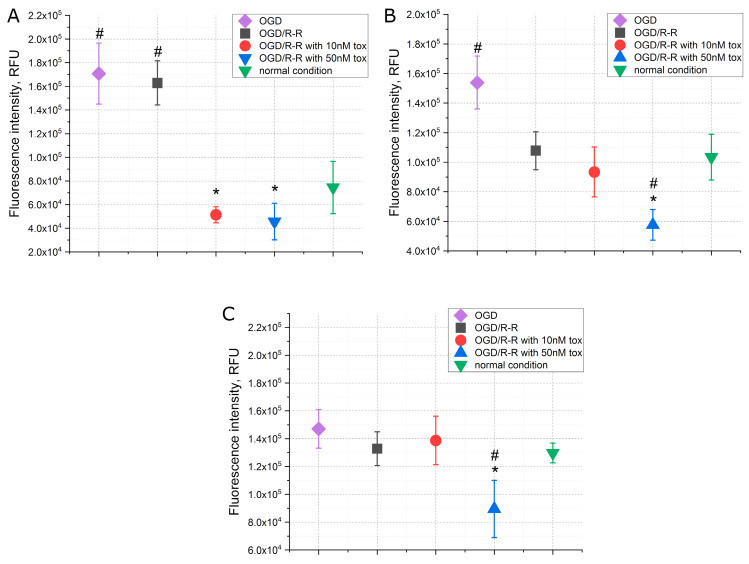
The concentrations of calcium (**A**), sodium (**B**), potassium (**C**) when simulating the OGD/R-R conditions in CHO-K1 culture with the addition of omega-hexatoxin-Hv1a toxin (OGD—oxygen–glucose deprivation; R-R—reoxygenation/reperfusion; RFU—relative fluorescence units). (*—*p* < 0.01 when compared with the “OGD/R-R” group, #—*p* < 0.01 when compared with the “normal condition” group).

The data on the concentration of calcium, sodium and potassium ions in the simulation of the OGD/R-R conditions presented in Figure 3 showed that, immediately after 3 h OGD, there was an increase in the concentrations of calcium and sodium, approximately 2.5 and 1.5 times, respectively, while the concentration of potassium did not change. At the reoxygenation–reperfusion stage, the concentration of calcium ions remained at the same elevated level (Figure 3A), and the concentration of sodium ions decreased to the level of normal conditions (Figure 3B). There was no change in potassium concentration during the experiment (Figure 3C). The addition of toxin at the reoxygenation–reperfusion stage, in the case of calcium ions, led to a decrease in the concentration below normal conditions, at 10 nM of the toxin by about 30%, at 50 nM by about 40%. In the case of sodium and potassium ions, in the presence of the toxin at a concentration of 50 nM, there was also an additional decrease in the concentrations below the normal level, by about 50% in the case of sodium ions and about 30% in the case of potassium ions. At a toxin concentration of 10 nM, the above was not observed.

**Figure 4:**
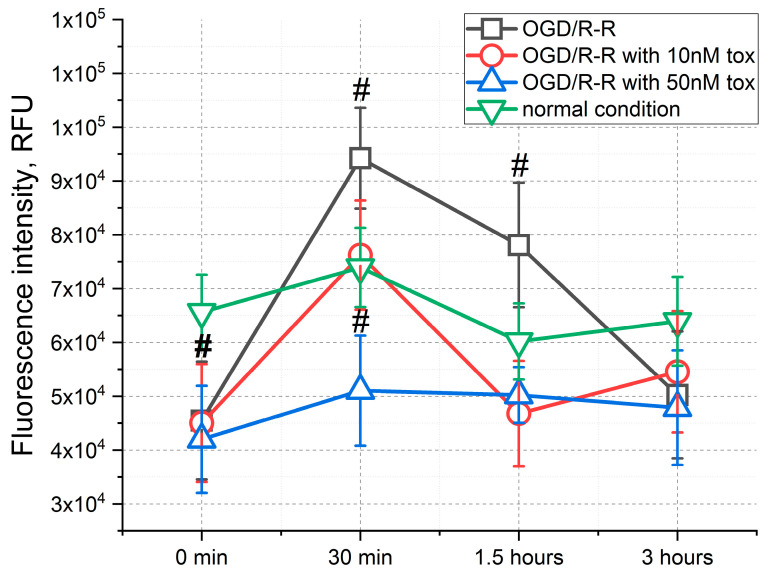
Changes in the pH level within 3 h from the start of reoxygenation-reperfusion when simulating the OGD/R-R conditions in CHO-K1 culture with the addition of omega-hexatoxin-Hv1a toxin (OGD—oxygen–glucose deprivation; R-R—reoxygenation/reperfusion; RFU—relative fluorescence units) (#—*p* < 0.01 when compared with the “normal condition” group).

Significant changes in the pH level in modeling the OGD/R-R conditions occurred (Figure 4). For example, immediately after the completion of the OGD, a drop in the pH of about 30% was noted. At the same time, in the next 30 min, the pH level rose to about 30% above normal conditions. This was followed by a gradual decrease in the pH to a normal level. The addition of toxin in this case was dose-dependent. So, at a toxin concentration of 10 nM, an increase in the pH was also recorded in the first 30 min, but the increase was only limited by about 10%, followed by keeping at this level. Additionally, if there was a toxin in the medium at a concentration of 50 nM, the pH level slowly rose to the normal level during the entire reoxygenation–reperfusion stage for 3 h.

**Figure 5:**
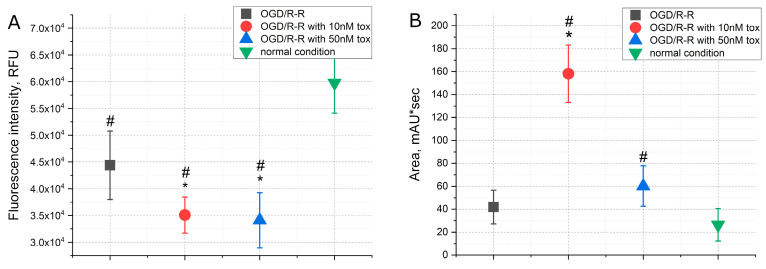
The level of mitochondrial potential (**A**) and ATP (**B**) when simulating OGD/R-R conditions in CHO-K1 culture with the addition of omega-hexatoxin-Hv1a toxin (OGD—oxygen–glucose deprivation; R-R—reoxygenation-reperfusion; RFU—relative fluorescence units). (*—*p* < 0.01 when compared with the “OGD/R-R” group, #—*p* < 0.01 when compared with the “normal condition” group).

From the data on the level of mitochondrial potential and the concentration of ATP presented in Figure 5, it can be seen that the mitochondrial potential decreased by about 25% during the simulation of the OGD/R-R conditions (Figure 5A). The addition of the toxin in this case further reduced the mitochondrial potential; at toxin concentrations of 10 nM and 50 nM, the potential level was reduced by about 20%. In addition, despite the fact that the ATP concentration did not change during the reoxygenation–reperfusion stage, the toxin at a concentration of 10 nM caused a significant increase in ATP, approximately six times relative to normal conditions (Figure 5B). At a concentration of 50 nM, such an increase in the ATP concentration was moderate, approximately two times.

**Figure 6:**
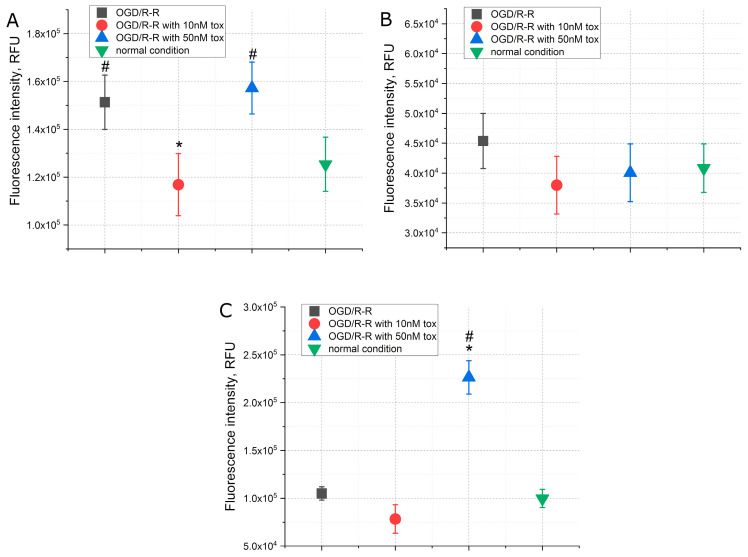
The concentrations of reactive oxygen species (**A**), nitric oxide II (**B**), glutathione (**C**) when simulating the OGD/R-R conditions in CHO-K1 culture with the addition of omega-hexatoxin-Hv1a toxin (OGD—oxygen–glucose deprivation; R-R—reoxygenation-reperfusion; RFU—relative fluorescence units). (*—*p* < 0.01 when compared with the “OGD/R-R” group, #—*p* < 0.01 when compared with the “normal condition” group).

The antioxidant system also underwent changes when the OGD/R-R conditions were simulated. Similar changes manifested themselves in an increase in reactive oxygen species (ROS). Thus, the concentration of ROS increased by about 1.5 times during reoxygenation–reperfusion (Figure 6A). However, the concentrations of nitric oxide II and glutathione were not affected (Figure 6B,C). The addition of toxin at a concentration of 10 nM in this case prevented the increase in the concentration of reactive oxygen species (Figure 6A) without affecting nitric oxide II and glutathione (Figure 6B,C). On the other hand, the toxin at a concentration of 50 nM did not affect the concentration of reactive oxygen species and nitric oxide II, but caused an increase in glutathione by about two times.

Thus, when simulating the OGD/R-R conditions in the CHO-K1 culture, an increase in the level of apoptosis and necrosis was observed, as well as an increase in the concentration of calcium ions and reactive oxygen species, while the concentration of sodium ions and the mitochondrial potential decreased. In addition, the pH value rose sharply in the first 30 min from the start of reoxygenation–reperfusion and decreased to a normal level within 2.5 h. The concentrations of potassium ions, ATP, nitric oxide II and glutathione did not change. The addition of toxin to the medium for reoxygenation–reperfusion at a concentration of 10 nM led to a decrease in necrosis, a faster normalization of the cellular index, and caused a decrease in the concentration of calcium ions, without affecting sodium and potassium ions. The pH level rose to a normal level in the first 30 min and maintained at that level until the end of reoxygenation–reperfusion. In addition, when the toxin was added at a concentration of 10 nM, the mitochondrial potential and the concentration of reactive oxygen species decreased, but ATP synthesis increased. When the toxin was added at a concentration of 50 nM, in addition to reducing necrosis, apoptosis was also reduced. At this concentration, the concentration of calcium ions also decreased; however, a decrease in the concentration of sodium and potassium was also recorded. The pH value slowly normalized during the entire reoxygenation–reperfusion stage. The mitochondrial potential also decreased (this was also observed at a concentration of 10 nM); however, in this case, ATP synthesis was more moderate. With the addition of 50 nM toxin, the concentration of reactive oxygen species and nitric oxide II did not change compared to the control conditions, but the concentration of glutathione increased.

## 3. Discussion

Numerous studies have shown that calcium is an important regulator of cell death both at the stage of ischemia and at the stage of reperfusion. Thus, an increase in intracellular calcium occurs at the stage of ischemia and becomes even more pronounced during reperfusion. In an attempt to cope with the considerable changes in cytosolic Ca^2+^ levels, transport through the mitochondrial Ca^2+^ uniporter is increased. Using a negative membrane potential, this transporter controls the movement of positively charged Ca^2+^ ions into the mitochondria. On the one hand, there is a decrease in cytosolic Ca^2+^; on the other hand, an increase in mitochondrial Ca^2+^ takes place—all this triggers the opening of the transitional mitochondrial permeability pore. Pathological activation of calpains, a family of cysteine proteases targeting various cytoskeletal, ER, and mitochondrial proteins, as well as suppression of the activity of Ca^2+^/calmodulin-dependent protein kinases, also occurs in response to an induced increase in Ca^2+^ [21,22,23]. The level of calcium in the cytosol is regulated by calcium-permeable ion channels localized either on the membranes of specific intracellular organelles or on the plasma membrane. This group also includes L-type voltage-gated calcium ion channels. We propose to reduce the concentration of calcium ions during the reperfusion stage using omega-hexatoxin-Hv1a. An important feature of the omega-hexatoxin-Hv1a toxin is its selectivity for voltage-gated L-type calcium ion channels. According to the mechanism of action, the toxin belongs to the blockers of pore channels [24]. 

In this work, the effect of omega-hexatoxin-Hv1a on intracellular processes in the CHO-K1 culture is studied through modeling the OGD/R-R conditions. The tissues of the cardiovascular and nervous systems are known to be most vulnerable to IRI [1,2]. However, a number of studies have shown that cells of epithelial origin, e.g., the epithelium of the tubules of the kidneys, as well as the epithelial cells of the lung, are also significantly susceptible to IRI [25,26]. The CHO-K1 culture belongs to epithelial cells endogenously expressing voltage-gated L-type calcium channels [27] and can serve as a basis for studying the mechanisms of prevention of IRI in tissues and organs.

In our previous work, we have demonstrated the antiapoptotic effect of omega-hexatoxin-Hv1a toxin during peptide-induced apoptosis in CHO-K1 cells [28]. In this paper, we show that the mechanism of action of the toxin is different.

In our present experiments, the development of the IRI is recorded according to canonical mechanisms. An increase in the level of apoptosis and necrosis is noted, accompanied by a decrease in cell adhesion (cell index), mitochondrial potential and an increase in the concentration of reactive oxygen species (Figure 2A,B, Figure 5A and Figure 6A). However, when the toxin is added at a concentration of 50 nM at the stage of reperfusion, the level of apoptosis and necrosis, although increased, is less pronounced, while the cell index returns to normal much faster. Despite this, the level of mitochondrial potential in the presence of toxin in the medium for reperfusion further decreases, and ATP synthesis increases (Figure 5A,B). The concentration of active forms decreases with the addition of 10 nM toxin, and at 50 nM, it does not differ from the experimental group. However, the concentration of glutathione increases at 50 nM of toxin (Figure 6A,C).

A change the ion concentration and a decrease in the pH level are known to be the major effects during ischemia [29]. The concentration of sodium ions increases during ischemia and quickly returns to the preischemic state during reperfusion, while the concentration of calcium ions increases during ischemia and continues to grow during reperfusion until the activation of the mechanisms of cell death [30]. This effect has been observed in our experiments: Immediately after ischemia, an increase in the concentrations of calcium and sodium ions was recorded (Figure 3A,B). After that, at the stage of reperfusion, in addition to an increase in the level of apoptosis and necrosis (Figure 2), a high concentration of calcium ions and a reduced concentration of sodium ions were noted. In this case, the addition of 50 nM toxin leads to a significant decrease in the concentration of calcium and sodium ions at the stage of reperfusion (Figure 3A,B), which correlates with the changes in the level of apoptosis and necrosis (Figure 2). In our case, the mechanism of preventing cell death during the development of IRI by omega-hexatoxin-Hv1a toxin can be related to the direct blocking of the entry of extracellular calcium ions into the cell, which thereby prevents an increase in the calcium concentration at the reperfusion stage. The reduced calcium concentration, in turn, prevents cell death by the mechanism of apoptosis and necrosis. A similar effect is observed with the action of the L-type calcium channel blocker benidipine. Studies show that benidipine reduces apoptosis and necrosis when modeling conditions of ischemia–reperfusion in experiments in vivo [31,32]. A similar effect is manifested by the action of lacidipine, a blocker of slow calcium channels from the group of dihydropyridine derivatives, when ATP is depleted in the epithelial cells of the renal tubules [33], and also when adding verapamil to ischemic intestinal epithelial cells. In the latter case, the activation of the mechanisms of cell death is associated with a change in the distribution of integrins and depolymerization of F-actin [34]. However, in all cases, significant concentrations of the active substance are added to achieve the effect. In our study, a significant effect is already observed at a toxin concentration of 10 nM.

In addition to changes in ion concentration, the pH level plays a significant role in the development of IRI. As described above, during ischemia, a decrease in the pH occurs due to the accumulation of H^+^ and the inability of the cell to equalize the proton gradient. At the stage of reperfusion, with normalization of nutrient content and return to a normal pH, increased cell death is noted [35]. Research shows that manipulations associated with delaying the pH level normalization can prevent cell death [36]. In our case, there was a decrease in the pH level immediately after ischemia. During subsequent reperfusion, the pH rose above normal for the first 30 min and then slowly decreased to normal. The addition of toxin at a concentration of 10 nM in this case caused the pH to rise to normal in the first 30 min and maintained at this level until the end of reperfusion. When the toxin was added at a concentration of 50 nM, the pH was maintained below normal for 1.5 h (Figure 4) and reached normal after 3 h. Hence, in addition to reducing the concentration of calcium ions, the toxin indirectly affected the change in pH, slowing down its increase and delaying rapid normalization. 

In our previous work, we have already investigated the effect of the toxin modulator of sodium voltage-gated channels, mu-agatoxin-Aa1a, on CHO-K1 cell culture under IRI conditions [37]. Both studies have shown that both the sodium channel modulator toxin and the calcium channel blocker toxin reduce condition-induced IRI cell death, but the mechanism of action is different. Thus, the sodium channel modulator reduces the level of apoptosis and necrosis by maintaining the intracellular pH at an elevated level during the entire stage of reoxygenation–reperfusion, and also prevents a decrease in the concentration of sodium ions, while the calcium channel blocker provides a protective mechanism through the slow normalization of the intracellular pH during the entire stage of reoxygenation–reperfusion, which is accompanied by a decrease in the concentration of calcium and sodium ions. This allows us to evaluate the effect of both calcium ions and sodium ions on the development of IRI in epithelial cells.

In conclusion, the study has demonstrated that omega-hexatoxin-Hv1a toxin from the venom of the spider *Hadronyche versuta*, which belongs to the group of blockers of L-type voltage-gated calcium channels, is able to prevent mass death of cells of epithelial origin during the development of IRI. The mechanism of action of the toxin is associated with a decrease in the concentration of calcium ions at the stage of reperfusion, as well as with the prevention of a rapid normalization of the pH level. The strategy of using calcium blockers as molecules to reduce IRI is promising. Omega-hexatoxin-Hv1a can be used as an alternative to chemical calcium channel blockers to prevent IRI. When used in clinical practice, the toxin can be considered as a new class of drugs that enables cells and tissues to adapt to a rapidly changing metabolism during reperfusion, for example, in balloon angioplasty [38,39].

## 4. Materials and Methods

1. Peptide synthesis, quality control and formation of the secondary structure of toxin

Omega-hexatoxin-Hv1a toxin (amino acid sequence: SPTCIPSGQPCPYNENCCSQSCTFKENENGNTVKRCD) (UniProt ID: TO1A_HADVE) was used in the experiment as a blocker of calcium ion channels. Peptide synthesis was carried out with the ResPep SL automated peptide solid-phase synthesizer (Intavis, Tübingen, Germany). The synthesis was carried out on TentaGel resin (Intavis, Germany) (10 mg) together with Fmoc-L-aminoacid (Intavis, Germany). 0.5 M HBTU (2-(1H-benzotriazol-1-yl)-1,1,3,3-tetramethyluronium hexafluorophosphate) (Chemical line, Saint Petersburg, Russia) was used as an activator. Fmoc-group was deprotected with 10% N-methylpiperidine (Acros Organics, Geel, Belgium) in dimethylformamide (Chemical line, Russia) for 15 min. Capping was carried out with 5% acetic anhydride (Sigma Aldrich, St. Louis, MO, USA). The peptide was cleaved from its solid support with a solution of 92.5% trifluoroacetic acid (PanReac AppliChem, Barcelona, Spanish), 5% triisopropyl silane (Acros Organics, Belgium) and 2.5% water (3 h, room temperature) for 3 h. The peptide in solution was then precipitated in 3 × 500 µL of cold methyl tert-butyl ether (Chemical line, Russia). Toxin was dissolved in water and dried by lyophilization, then dissolved in folding buffer. The folding buffer contained 10 mM reduced glutathione and 1 mM oxidized glutathione in 0.1 M Tris-HCl (PanEco, Moscow, Russia), pH = 8.0 at 4 °C with gentle rocking for 24 h [40].

Shimadzu LC-20AD XR chromatographic system (SPD-20A detector) (Shimadzu, Kyoto, Japan) was used for peptide analysis by reverse phase chromatography using a Dr. Maisch Luna C18 column according to the standard protocol of gradient elution from 95% A (deionized water) and 5% B (acetonitrile (Cryochrom, Saint Petersburg, Russia)), followed by an increase in the concentration of eluent B to 100% for 40 min. MALDI-TOF MS with Flex Control 3.4 software (Bruker Daltonics, Bremen, Germany) was used for mass spectrometric analysis. HPLC (NGC Quest ™ 10 chromatography system (Bio-Rad, Hercules, CA, USA) was used for purification of toxin with Bio-Gel P-4 sorbent on an Econo-Column 1 × 30 cm column (Bio-Rad, USA).

2. Cell culture and experiment condition

The study was carried out on the Chinese hamster cells (CHO-K1 line) (Russian cell culture collection of Vertebrates, Saint Petersburg, Russia). The cell line was kept in DMEM (PanEco, Russia), supplemented with 10% FBS (Biosera, Cholet, France) and gentamicin at a final concentration of 80 μg/mL at 37 °C, 95% and 5% CO_2_ in CO_2_ incubator MCO-5AC (Sanyo, Osaka, Japan). Cells were seeded in 96-well plates (SPL Life Sciences, Pochon, Republic of Korea) at a concentration of 10,000 cells per well to reach exponential stage. 

To reproduce the model IRI, the cell culture was incubated for 3 h in DMEM medium with 1% of FBS and 1 g/L glucose in 1% O_2_ and 5% CO_2_ (oxygen–glucose deprivation) in a CB-53 incubator (Binder, Tuttlingen, Germany), followed by incubation for 3 h in DMEM with 10% FBS and 3.151 g/L glucose with 18.6% O_2_ and 5% CO_2_ (reoxygenation–reperfusion). The omega-hexatoxin-Hv1a at concentrations of 10 nM and 50 nM was added at the stage of reoxygenation–reperfusion. The control group of the cells were incubated under normal conditions in medium DMEM with 10% FBS and 3.151 g/L glucose with 18.6% O_2_, 5% CO_2_. Before the start of each experiment, the media were equilibrated for 30 min under the necessary conditions [41].

3. Measuring the level of apoptosis, necrosis, mitochondrial membrane potential, concentration of ROS, NO, ATP, GSH, calcium, sodium, potassium ions, pH and cell index

Changes in the level of apoptosis (Yo-Pro 1, 1 μM) [42], necrosis (PI, 1 μM) [42], the mitochondrial membrane potential (TMRE, 1 μM) [43], concentration of ROS (DCFH DA 1 μM), [43] NO (DAF-FM DA 1 µM) [44], GSH (MCB 1 µM) [45] calcium (Rhod-2 AM 500 nM) [46], sodium (ION NaTRIUM Green-2 AM, 500 nM) [47] and potassium (ION Potassium Green-2 AM, 500 nM) [48] ions were recorded. All dyes were added 3 h after the stage of reoxygenation and reperfusion and before it began for sodium, sodium and potassium ions. Changes in pH were also recorded throughout the reoxygenation and reperfusion stages at 0 min, 30 min, 1.5 h and 3 h using BCECF DA dye (1 μM) [49].

All dyes were added to cells and incubated for 20 min at 37 °C in the dark. After staining, medium was removed and all wells were washed twice with warm PBS. A multi-mode microplate reader CLARIO star Plus (BMG LABTECH, Ortenberg, Germany) was used for measurement of parameters. The measurement was carried out in 100 µL of PBS in the matrix scan mode (10 × 10). After the experiment, cell concentration was calculated. Primary data processing was carried out in the MARS program (BMG LABTECH, Germany) with subsequent processing in Excel. All data were recalculated per 100,000 cells.

To detect changes in the cell index, we used an xCellingence RTCA-S16 cell analyzer (ACEA Biosciences, San Diego, CA, USA) [50]. The cells were seeded at a concentration of 10,000 cells per well of a 16-well plate. The cell index was recorded in real time with an interval of 3 h under normal conditions at 37 °C and 5% CO_2_ in an MCO-5AC incubator (Sanyo, Japan). When the exponential stage of cell growth was reached, the medium in the cells was replaced by OGD medium, and the cells were transferred to an incubator with 1% O_2_ and 5% CO_2_. Change of cell index was carried out with an interval of 30 min for 3 h. After 3 h, the medium was replaced with a normal medium with the addition of toxin at different concentrations and the cells was placed in an incubator with 18.6% O_2_, 5% CO_2_ at 37 °C and the measurement continued. At the end of the measurement, the data were processed in Excel.

4. ATP analysis

For ATP analysis, cells were lysed in a buffer containing 0.02 M glycine, 0.05 M Mg^2+^, 0.004 M EDTA (pH = 7.4 at 25 °C) and heated in a water bath (100 °C) for 45 s.

By anion ion exchange liquid chromatography using an Agilent PL-SAX 4.6 × 150 mm column (PL1551-3802) on Shimadzu LC-20AD XR chromatographic system equipped with an SPD-20A spectrophotometric detector was determined of the relative ATP concentration. The analysis was carried out according to the following protocol: 0–2 min—100% A, 2–10 min—linear gradient 0–100% B, 10–14 min—100% B. Where A—deionized water, B—1 M NaCl, a wavelength of 257 nm [51].

5. Statistics

Each experiment was performed three times in three repetitions. For determine the nature of the distribution asymmetry and kurtosis criteria were used. To assess the statistical significance of differences the Mann–Whitney test was used (due to a small sample size); processing was performed in the Origin software (OriginLab, Northampton, MA, USA). Since the toxin exposure data were compared with the control conditions without a toxin, as well as with normal conditions, the Bonferroni test was used to eliminate the effect of multiple comparisons, and differences between groups were considered statistically significant at *p* ≤ 0.01.

## Data Availability

Data is contained within the article.
